# MicroRNAs and extracellular vesicles in the gut: new host modulators of the microbiome?

**DOI:** 10.1093/femsml/uqab010

**Published:** 2021-08-10

**Authors:** Xiaochen Du, Ruth Ley, Amy H Buck

**Affiliations:** Institute of Immunology & Infection Research, School of Biological Sciences, University of Edinburgh, Edinburgh EH9 3FL, UK; Department of Microbiome Science, Max Planck Institute of Developmental Biology, 72026 Tuebingen, Germany; Institute of Immunology & Infection Research, School of Biological Sciences, University of Edinburgh, Edinburgh EH9 3FL, UK

**Keywords:** extracellular vesicle, cross-species, microRNA, microbiome, extracellular RNA, RNA therapeutics

## Abstract

The gut microbiota plays an integral role in human health and its dysbiosis is associated with many chronic diseases. There are still large gaps in understanding the host and environmental factors that directly regulate the gut microbiota, and few effective strategies exist to modulate the microbiota in therapeutic applications. Recent reports suggest that certain microRNAs (miRNAs) released by mammalian cells can regulate bacterial gene expression to influence the microbiome composition and propose extracellular vesicles as one natural mechanism for miRNA transport in the gut. These new findings interface with a burgeoning body of data showing that miRNAs are present in a stable form in extracellular environments and can mediate cell-to-cell communication in mammals. Here, we review the literature on RNA-mediated modulation of the microbiome to bring cross-disciplinary perspective to this new type of interaction and its potential implications in biology and medicine.

## Teaser

Review and synthesis on current thinking and gaps in knowledge on how mammalian microRNAs and extracellular vesicles can directly modulate the gut microbiome.

## THE GUT MICROBIOTA AND ITS MODULATION

The mammalian gastrointestinal (GI) tract hosts a complex and diverse population of microorganisms collectively known as the gut microbiota. The number of microorganisms inhabiting the GI tract has been estimated to be 10^13^–10^14^ and the gut microbiome contains over 100 times as many genes as the human genome (Thursby and Juge 2017). The gut microbiota contributes to host health through various physiological functions such as maintaining intestinal barrier (Natividad and Verdu 2013), modulating the host's energy metabolism (Cani *et al*. 2019), protecting against infection by pathogenic bacteria (Buffie *et al*. 2015) and shaping host immunity (Wu *et al*. 2010; Gaudet *et al*. 2015). Many studies suggest that the maintenance of gut bacterial community structure is important for health and that dysbiosis of the gut microbiota is associated with numerous human diseases, including inflammatory bowel diseases (Nishino *et al*. 2018), obesity (Walters, Xu and Knight 2014), diabetes mellitus (Wen *et al*. 2008) and GI cancers (Serban 2014).

There is a growing need for new approaches to specifically manipulate the gut microbiota, and there is also increasing interest in understanding the natural host mechanisms involved in its regulation. It is well known that host-derived products, such as mucus, antimicrobial peptides and IgA, produced by the intestinal epithelial cells, can encourage the growth of some microbial species and inhibit that of others (Suzuki *et al*. 2004; Carvalho *et al*. 2012). In the last 5 years, several papers have shown that mammalian microRNAs (miRNAs) can influence the composition and activity of the gut bacteria in a specific way, for example through direct RNA–RNA interactions with bacterial genes. These findings interface with a burgeoning body of literature showing that small RNAs, including miRNAs, can operate beyond the cell in which they derive in mammals, through transport in extracellular vesicles (EVs) or other carriers.

## miRNAs: FUNCTION AND REGULATION IN THE GUT

There are three predominant classes of small RNA in eukaryotes that mediate gene regulation and genome defence: small interfering RNAs, PIWI-associated RNAs and miRNAs (Kutter and Svoboda 2008). Of these classes, miRNAs are the most well studied in mammals for their roles in development and disease (Kloosterman and Plasterk 2006). miRNAs derive from endogenous genes that are typically transcribed by RNA polymerase II into primary miRNA transcripts that undergo processing by Drosha and other cofactors in the nucleus (Pasquinelli 2012). This processing produces the pre-miRNAs (∼60–70 nucleotides in length) that are transported to the cytoplasm by Exportin 5 where they are further processed by Dicer, which removes the loop region from pre-miRNAs. One strand of the resulting duplex is bound by an Argonaute (Ago) protein to form the RNA-induced silencing complex (RISC). Within RISC, the miRNA serves as the guide to bind to specific messenger RNA targets through sequence complementarity and this results in either cleavage of the mRNA or inhibition of translation (Bartel 2018).

Many reports have now demonstrated the importance of miRNAs in gut function. For example, fewer goblet cells, impaired nutrient absorption and barrier function were observed in mice in which Dicer was ablated from intestinal epithelial cells (McKenna *et al*. 2010). The fate of human intestinal epithelial cells was also shown to be directly regulated by transfection of synthetic miRNA mimics or inhibitors (Dalmasso *et al*. 2010). Moreover, miRNA expression profiles within intestinal cells have been shown to change during various intestinal diseases, and some of the miRNAs are likely to be causative agents in disease progression and development. For example, the level of miR-21 increased in colonic tissue from active ulcerative colitis and Crohn's disease, and its pathogenic role was confirmed in miR-21 knockout mice (Wu *et al*. 2008; Shi *et al*. 2013; Yang *et al*. 2013).

miRNAs also play a role in the ability of intestinal cells to sense and respond to changes in the environment. For example, intestinal miRNA levels are modulated when exposed to high amounts of dietary lipids (Gil-Zamorano *et al*. 2020), and the presence or absence of the microbiome directly affects miRNA expression in intestinal cells (Dalmasso *et al*. 2011; Singh *et al*. 2012). miRNA expression levels were further shown to be altered in a highly cell type-specific manner, with the strongest deregulation in stem cells, suggesting important roles of miRNAs in regulating intestinal homeostasis (Peck *et al*. 2017).

## EXTRACELLULAR miRNAs AND RNA COMMUNICATION IN THE GUT

The above work suggests that the biogenesis and function of miRNAs may be closely tied to sensing changes in the intestinal environment. At the same time, miRNAs are also now recognized to be released into the environment, with scope to act outside of the cells in which they derive. Extensive literature in the last 15 years has shown that miRNAs can be detected in a cell-free form in different body fluids (Murillo *et al*. 2019). Furthermore, miRNAs are stable and can be reliably detected in faeces from human and mice (Ahmed *et al*. 2009; Wu *et al*. 2012), providing a noninvasive method to sample gut luminal miRNAs in different contexts. For example, the levels of miR-21 and miR-92a were significantly higher in the stool of patients with colorectal cancer compared with healthy controls (Wu *et al*. 2012). There is an increasing interest in using faecal miRNAs as noninvasive markers of intestinal malignancies (Yau *et al*. 2019).

Beyond their biomarker potential, miRNAs and other classes of extracellular small RNA have also been reported to participate in communication between different organisms and species (Claycomb, Abreu-Goodger and Buck 2017). The relative abundance and stability of mammalian miRNAs in the gut lumen therefore lead to the question of whether these could modulate gene targets in other organisms in the gut.

## EVIDENCE OF DIRECT miRNA–BACTERIA INTERACTIONS IN THE GUT

Several recent studies have reported direct RNA–RNA interactions between host miRNAs and bacterial genes in the gut (Table 1), based on a combination of *in vivo* genetic experiments in mice and *in vitro* validation experiments.

**Table 1.** Reported miRNA–bacteria interactions in the gut.

miRNA	Bacteria species	Target transcript	Effect on bacteria	Reference
miR-1226-5p	*Escherichia coli*	yegH mRNA	Promotion	Liu *et al*. (2016)
miR-515-5p	*Fusobacterium nucleatum*	16S rRNA	Promotion	Liu *et al*. (2016)
miR-30d	*Akkermansia muciniphila*	β-Galactosidase mRNA	Promotion	Liu *et al*. (2019)
miR-21	*Lactobacillus reuteri*	Unknown	Inhibition	Santos *et al*. (2020)

Liu *et al*. (2016) used Dicer knockout mice in a first demonstration that the deficiency of miRNAs in intestinal epithelial cells caused an imbalanced gut microbiota, which can then be restored by transplantation of wild-type faecal miRNAs (where the native RNA is extracted from faeces and administrated to mice by gavage). To explore whether specific miRNAs can directly interact with gut bacteria, the authors selected two important gut bacteria, *Fusobacterium nucleatum* and *Escherichia coli*, to predict their potential interacting miRNAs by sequence similarity. By culturing the strains with synthesized miRNAs *in vitro*, they found that miRNA-515-5p can stimulate *F. nucleatum* growth and that miRNA-1226-5p can stimulate *E. coli* growth. The expression levels of predicted miRNA targets in these two strains were also found to be increased in response to the synthetic RNA. In a subsequent study, the same authors showed that miR-30d, which is enriched in the faeces of animals with experimental autoimmune encephalomyelitis (EAE), can promote the growth of *Akkermansia muciniphila* by increasing the expression of β-galactosidase (Liu *et al*. 2019). These examples suggest that host miRNA interactions with bacterial transcripts can lead to an increase in bacterial gene expression and bacterial growth. A more recent study suggests that miRNAs can also suppress bacterial growth. In particular, the composition of gut microbiota in miR-21 knockout mice was characterized by an increase in *Lactobacillus* and incubation of synthetic miR-21 with *Lactobacillus reuteri* led to reduced growth (Santos *et al*. 2020).

## IMPLICATIONS AND QUESTIONS

**Figure 1.**
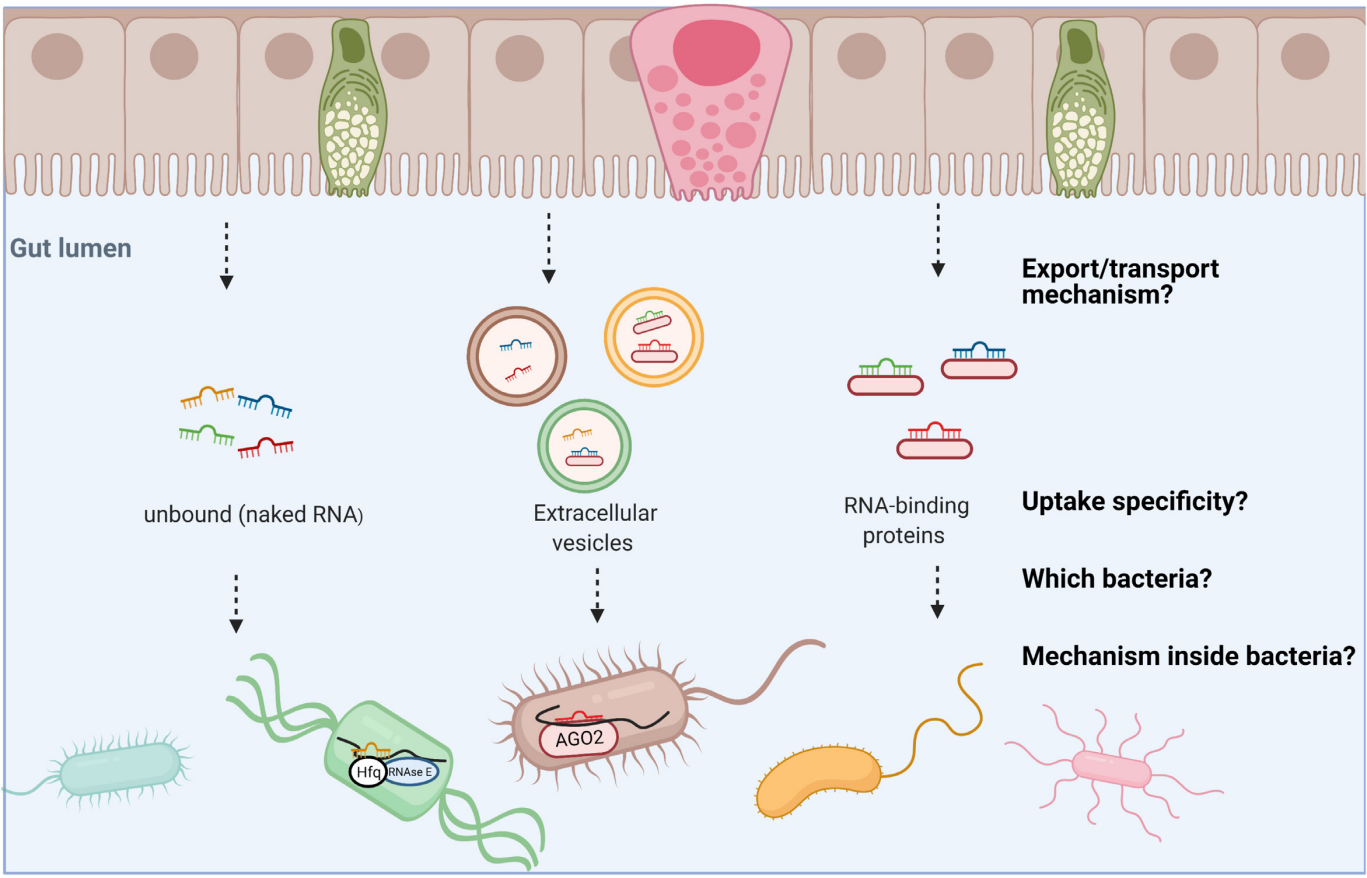
Knowledge gaps in miRNA–bacteria interactions.

The above studies suggest that specific mammalian miRNAs are naturally transported into some bacterial cells, where the miRNA forms a direct RNA–RNA interaction with a bacterial gene, resulting in changes in the microbiome with phenotypic effects in intestinal health. The potential impact of these findings is immense, since this suggests that intestinal miRNAs are an integral part of how the microbiome is regulated. Furthermore, since it is easy to synthesize different RNA sequences, one could envision this as a new, programmable method to dictate changes to the composition or activity of the microbiome. Yet some big questions remain in terms of how miRNA–bacteria interactions are specified and controlled, how the RNA–RNA interactions evolve and, ultimately, whether and how synthetic RNA is a viable and safe strategy for microbiome manipulation (Fig. 1). Here, we attempt to discuss these questions and provide some directions for future study.

## APPROACHES TO IDENTIFY SPECIFIC miRNA–BACTERIA INTERACTIONS

Since there are thousands of miRNAs as well as bacterial species/strains in the gut, it is a daunting prospect to systematically determine or predict which miRNA–bacteria interactions occur. The strategy used by Liu *et al*. (2019) was to identify miRNAs that are altered in faeces in different disease contexts and to link this to changes in the microbiome. For example, *A. muciniphila* was found to be increased in the faeces from EAE mice compared with controls, and miR-30d was also enriched in faeces from EAE mice (Liu *et al*. 2019). However, one could imagine a lot of false positives in this approach, and it should be noted that the level of a miRNA in faeces is not a direct proxy for miRNAs internalized by bacteria, since dead host cells and extracellular luminal miRNAs will also be present. Nonetheless, Liu *et al*. (2019) showed that miR-30d could directly impact the growth of *A. muciniphila* by culturing the strain in the presence of synthetic miR-30d *in vitro* and also through oral administration of synthetic miR-30d to mice *in vivo*. The miRNA-dependent increase in bacterial growth was not observed using scrambled miRNA sequence controls (Liu *et al*. 2019).

miRNA–bacteria interactions can also be inferred based on studies in miRNA knockout mice, as demonstrated by Santos *et al*. (2020). In the miR-21 knockout mouse, *Lactobacillus* was found to be significantly increased in the small intestinal lumen when compared with wild-type animals (Santos *et al*. 2020). Although ablation of the miRNA could also indirectly affect the microbiome (e.g. through altered host cell populations and functions), the authors showed that synthetic miR-21 directly suppressed the growth of *Lactobacillus* by *in vitro* assays. Sequence specificity was demonstrated in this study too, since the scrambled miR-21 sequence did not show effects. Ultimately, understanding the host transport mechanisms of miRNAs could help shed light on whether and how there is specificity, for example, in which bacteria can internalize various extracellular miRNAs, and what machinery is required within the bacteria for the miRNAs to be functional.

## TRANSFER MECHANISM FOR miRNA INTO BACTERIA CELLS: A ROLE OF EXTRACELLULAR VESICLES?

The published studies described above have cultured specific bacteria with synthetic miRNAs *in vitro* to show that naked miRNAs can enter bacteria and uptake or activity is sequence specific. However, the natural miRNA uptake mechanism in the gut environment is not known, nor is it clear whether only specific bacteria internalize RNA.

In mammals, EVs are the most well-studied mechanism for miRNA stabilization and transfer between cells. There are three general classes of EVs, depending on their biogenesis: (i) exosomes (generally 50–100 nm in size), which are of endosomal origin and derive from multivesicular bodies; (ii) microvesicles (generally 100–1000 nm in size), which bud off the plasma membrane; and (iii) apoptotic bodies (generally 50–5000 nm in size), which are generated when cells undergo apoptosis (Colombo, Raposo and Théry 2014). Several key studies demonstrated that intestinal epithelial cells release exosomes, suggesting these are involved in antigen presentation (Van Niel *et al*. 2001, 2003). However, we now know that exosomes and other classes of EVs also contain RNAs, including miRNAs (Veziroglu and Mias 2020). Seminal work by Valadi *et al*. (2007) showed that miRNAs can be transported from one mast cell to another via exosomes. Microvesicles derived from tumours were shown to deliver miRNAs to CD4^+^ T cells and thereby increase the number of regulatory T cells, promoting tumour growth (Yin *et al*. 2014). EVs can also mediate communication between different species in the gut. For example, the GI nematode *Heligmosomoides polygyrus* releases miRNA-containing EVs that can be internalized by host mouse cells and modulate innate immunity during infection (Buck *et al*. 2014).

EVs therefore represent a potential transport mechanism for mammalian miRNAs in the gut, and there is some evidence that mammalian EVs interact with bacteria, with diverse functional outcomes. For example, EVs released from the respiratory epithelium during respiratory viral infection interact with *Pseudomonas aeruginosa* to provide nutrients and promote biofilms (Hendricks *et al*. 2021). EVs released from neutrophils or macrophages have also been shown to have antimicrobial activities (Timár *et al*. 2013; Garcia-Martinez *et al*. 2019). This work sets precedent that host EVs can impact bacteria and may directly transport host molecules into some species. Interestingly, although Liu *et al*. (2016) demonstrated that synthetic miRNAs can enter bacteria *in vitro* (without cofactors), they also showed that EVs containing miRNAs were present in human and mouse faecal samples. Yet the question of specificity remains: Do all bacteria internalize EVs? Are there ligand/receptor interactions that dictate which bacteria internalize these? Intriguingly, a recent paper showed that EVs from ginger plant can also transport miRNAs into *Lactobacillus rhamnosus*, and proposed that the lipid content of the EVs was important for uptake and potentially specificity (Teng *et al*. 2018). Given the ubiquity of EVs from organisms in the gut, as well as potential dietary sources of EVs, it will be important to test the breadth of cross-species transfer of miRNA by EVs and understand the underlying properties that define specificity. It remains possible that other types of gut inhabitants (e.g. fungi) could also internalize host miRNAs. There is little information of this type of interaction in mammalian systems but a burgeoning body of data suggesting plant–fungi RNA–RNA interactions (Cai *et al*. 2018).

The other more unexplored stabilization and transport mechanism for mammalian miRNAs is RNA-binding proteins. miRNAs have been reported to be delivered by high-density lipoprotein to recipient cells and to regulate the expression of target genes (Vickers *et al*. 2011). miRNAs are also found bound to Ago proteins in human plasma and serum, and this association is proposed to play a critical role in stabilizing miRNA (Arroyo *et al*. 2011; Turchinovich *et al*. 2011). Whether and how bacteria internalize host RNA–protein complexes remains unexplored.

## FUNCTIONAL INTEGRATION OF miRNAs IN BACTERIA

In mammals, it is assumed that miRNAs only function through association with an Ago protein, where their role is to guide this protein to a nucleic acid target with sequence complementarity. From a mechanistic standpoint, therefore, it is very relevant to understand whether Ago moves with miRNAs into bacteria. Several reports have demonstrated that Ago2 is transported into mitochondria, which is evolutionarily related to bacteria, and miRNA–Ago2 complexes can enhance mitochondrial translation (Zhang *et al*. 2014). Currently, there are no data on whether Ago proteins can enter bacteria in the gut, either in a vesicle-free form or within EVs.

However, it also remains possible that internalized miRNAs could interact with bacterial RNA-binding proteins. It is well known that bacteria use their own small RNAs to regulate gene expression and the best characterized mechanism involves the bacterial chaperone protein Hfq (Soper *et al*. 2010) that recruits the endoribonuclease RNase E (Morita and Aiba 2011). Since several reports have shown that culturing bacteria with synthetic miRNA mimics *in vitro* directly impacts bacterial gene expression (Liu *et al*. 2019; Santos *et al*. 2020), it may be important to consider whether and how miRNAs function with bacterial protein partners (Layton *et al*.^2020^).

## FUTURE PERSPECTIVE

Given the many diverse functions and mechanisms by which RNA can mediate cellular and organismal biology, it is an exciting prospect to consider RNA functions also in more complex living systems. In plants, mobile small RNAs have been shown to be part of a bidirectional arms race with fungal parasites (Weiberg *et al*. 2013; Cai *et al*. 2018) and plant sRNAs can also regulate genes in bacterial phytopathogens (Singla-Rastogi *et al*.^2019^). In honeybees, small RNAs are thought to mediate some of the caste-determining effects of royal jelly (Zhu *et al*. 2017) and are transmissible between organisms and across generations (Maori *et al*. 2019). As reviewed here, there are now several reports that mouse miRNAs can directly influence the growth rate of certain bacteria and thereby influence the composition and activity of the microbiome. These findings push forward the idea that RNA is central to cross-species interactions, and may implicate EVs as a ubiquitous enabler of RNA-based communication in the gut. Yet many questions remain regarding how the mammalian miRNA and bacterial target sites coevolve and what machinery is required (in each organism) for RNA communication. To date, only a handful of examples of host–bacteria RNA–RNA interaction exist, and these may be cherry-picked from a sea of possibilities. Ultimately, systematic analyses and mechanistic understanding are required to address the exciting questions of which organisms participate in RNA-mediated communication and how we can harness this mechanism in medicine.

## References

[bib1] Ahmed FE , Jeffries CD, Vos PW et al. Diagnostic microRNA markers for screening sporadic human colon cancer and active ulcerative colitis in stool and tissue. Cancer Genomics Proteomics. 2009;6:281–95. 19996134

[bib2] Arroyo JD , Chevillet JR, Kroh EM et al. Argonaute2 complexes carry a population of circulating microRNAs independent of vesicles in human plasma. Proc Natl Acad Sci USA. 2011;108:5003–8. 21383194 10.1073/pnas.1019055108 PMC3064324

[bib3] Bartel DP . Metazoan microRNAs. Cell. 2018;173:20–51. 29570994 10.1016/j.cell.2018.03.006 PMC6091663

[bib4] Bäumler AJ , Sperandio V. Interactions between the microbiota and pathogenic bacteria in the gut. Nature. 2016;535:85–93. 27383983 10.1038/nature18849 PMC5114849

[bib5] Buck AH , Coakley G, Simbari F et al. Exosomes secreted by nematode parasites transfer small RNAs to mammalian cells and modulate innate immunity. Nat Commun. 2014;5:5488. 25421927 10.1038/ncomms6488 PMC4263141

[bib6] Buffie CG , Bucci V, Stein RR et al. Precision microbiome reconstitution restores bile acid mediated resistance to *Clostridium difficile*. Nature. 2015;517:205–8. 25337874 10.1038/nature13828 PMC4354891

[bib7] Cai Q , He B, Weiberg A et al. Small RNAs and extracellular vesicles: new mechanisms of cross-species communication and innovative tools for disease control. PLoS Pathog. 2019;15:e1008090. 31887135 10.1371/journal.ppat.1008090 PMC6936782

[bib8] Cai Q , Qiao L, Wang M et al. Plants send small RNAs in extracellular vesicles to fungal pathogen to silence virulence genes. 2018;360:1126–9. 10.1126/science.aar4142 PMC6442475 29773668

[bib9] Cani PD , Van Hul M, Lefort C et al. Microbial regulation of organismal energy homeostasis. Nat Metab. 2019;1:34–46. 32694818 10.1038/s42255-018-0017-4

[bib10] Carvalho FA , Aitken JD, Vijay-Kumar M et al. Toll-like receptor–gut microbiota interactions: perturb at your own risk!. Annu Rev Physiol. 2012;74:177–98. 22035346 10.1146/annurev-physiol-020911-153330

[bib11] Claycomb J , Abreu-Goodger C, Buck AH. RNA-mediated communication between helminths and their hosts: the missing links. RNA Biol. 2017;14:436–41. 28125361 10.1080/15476286.2016.1274852 PMC5411118

[bib12] Colombo M , Raposo G, Théry C. Biogenesis, secretion, and intercellular interactions of exosomes and other extracellular vesicles. Annu Rev Cell Dev Biol. 2014;30:255–89. 25288114 10.1146/annurev-cellbio-101512-122326

[bib14] Dalmasso G , Nguyen HTT, Yan Y et al. Microbiota modulate host gene expression via microRNAs. PLoS One. 2011;6:e19293. 21559394 10.1371/journal.pone.0019293 PMC3084815

[bib13] Dalmasso G , Thu Nguyen HT, Yan Y et al. MicroRNAs determine human intestinal epithelial cell fate. Differentiation. 2010;80:147–54. 20638171 10.1016/j.diff.2010.06.005 PMC2943016

[bib15] Fisher K . MicroRNA in inflammatory bowel disease: translational research and clinical implication. World J Gastroenterol. 2015;21:12274–82. 26604636 10.3748/wjg.v21.i43.12274 PMC4649112

[bib16] Garcia-Martinez M , Vázquez-Flores L, Alvarez-Jiménez VD et al. Extracellular vesicles released by J774A.1 macrophages reduce the bacterial load in macrophages and in an experimental mouse model of tuberculosis. Int J Nanomed. 2019;14:6707–19. 10.2147/IJN.S203507 PMC6708438 31692512

[bib17] Gaudet RG , Sintsova A, Buckwalter CM et al. Cytosolic detection of the bacterial metabolite HBP activates TIFA-dependent innate immunity. Science. 2015;348:1251–5. 26068852 10.1126/science.aaa4921

[bib18] Gensollen T , Iyer SS, Kasper DL et al. How colonization by microbiota in early life shapes the immune system. 2016;352:539–44. 10.1126/science.aad9378 PMC5050524 27126036

[bib19] Gil-Zamorano J , Tomé-Carneiro J, Lopez De Las Hazas M-C et al. Intestinal miRNAs regulated in response to dietary lipids. Sci Rep. 2020;10:18921. 33144601 10.1038/s41598-020-75751-w PMC7642330

[bib20] Gottesman S , Storz G. Bacterial small RNA regulators: versatile roles and rapidly evolving variations. Cold Spring Harb Perspect Biol. 2011;3:a003798. 20980440 10.1101/cshperspect.a003798 PMC3225950

[bib21] Hendricks MR , Lane S, Melvin JA et al. Extracellular vesicles promote transkingdom nutrient transfer during viral-bacterial co-infection. Cell Rep. 2021;34:108672. 33503419 10.1016/j.celrep.2020.108672 PMC7918795

[bib22] Kloosterman WP , Plasterk RHA. The diverse functions of microRNAs in animal development and disease. Dev Cell. 2006;11:441–50. 17011485 10.1016/j.devcel.2006.09.009

[bib23] Kutter C , Svoboda P. miRNA, siRNA, piRNA: knowns of the unknown. RNA Biol. 2008;5:181–8. 19182524 10.4161/rna.7227

[bib62_1629379442699] Layton E , Fairhurst AM, Griffith-Jones S et al. Regulatory RNAs: A Universal Language for Inter-Domain Communication. Int J Mol Sci. 2020;21:8919. 33255483 10.3390/ijms21238919 PMC7727864

[bib24] Li X , Watanabe K, Kimura I. Gut microbiota dysbiosis drives and implies novel therapeutic strategies for diabetes mellitus and related metabolic diseases. Front Immunol. 2017;8:1882. 29326727 10.3389/fimmu.2017.01882 PMC5742320

[bib25] Liu S , da Cunha AP, Rezende RM et al. The host shapes the gut microbiota via fecal microRNA. Cell Host Microbe. 2016;19:32–43. 26764595 10.1016/j.chom.2015.12.005 PMC4847146

[bib26] Liu S , Rezende RM, Moreira TG et al. Oral administration of miR-30d from feces of MS patients suppresses MS-like symptoms in mice by expanding *Akkermansia muciniphila*. Cell Host Microbe. 2019;26:779–94. 31784260 10.1016/j.chom.2019.10.008 PMC6948921

[bib27] Maori E , Garbian Y, Kunik V et al. A transmissible RNA pathway in honey bees. Cell Rep. 2019;27:1949–59. 31056439 10.1016/j.celrep.2019.04.073

[bib28] McKenna LB , Schug J, Vourekas A et al. MicroRNAs control intestinal epithelial differentiation, architecture, and barrier function. Gastroenterology. 2010;139:1654–64. 20659473 10.1053/j.gastro.2010.07.040 PMC3156097

[bib29] Morita T , Aiba H. RNase E action at a distance: degradation of target mRNAs mediated by an Hfq-binding small RNA in bacteria. Genes Dev. 2011;25:294–8. 21325130 10.1101/gad.2030311 PMC3042153

[bib30] Murillo OD , Thistlethwaite W, Rozowsky J et al. exRNA Atlas analysis reveals distinct extracellular RNA cargo types and their carriers present across human biofluids. Cell. 2019;177:463–77. 30951672 10.1016/j.cell.2019.02.018 PMC6616370

[bib31] Natividad JMM , Verdu EF. Modulation of intestinal barrier by intestinal microbiota: pathological and therapeutic implications. Pharmacol Res. 2013;69:42–51. 23089410 10.1016/j.phrs.2012.10.007

[bib32] Nishino K , Nishida A, Inoue R et al. Analysis of endoscopic brush samples identified mucosa-associated dysbiosis in inflammatory bowel disease. J Gastroenterol. 2018;53:95–106. 28852861 10.1007/s00535-017-1384-4

[bib33] Pasquinelli AE . MicroRNAs and their targets: recognition, regulation and an emerging reciprocal relationship. Nat Rev Genet. 2012;13:271–82. 22411466 10.1038/nrg3162

[bib34] Peck BCE , Mah AT, Pitman WA et al. Functional transcriptomics in diverse intestinal epithelial cell types reveals robust microRNA sensitivity in intestinal stem cells to microbial status. J Biol Chem. 2017;292:2586–600. 28053090 10.1074/jbc.M116.770099 PMC5314158

[bib35] Santos AA , Afonso MB, Ramiro RS et al. Host miRNA-21 promotes liver dysfunction by targeting small intestinal *Lactobacillus*in mice. Gut Microbes. 2020;12:1–18. 10.1080/19490976.2020.1840766 PMC7733982 33300439

[bib36] Serban DE . Gastrointestinal cancers: influence of gut microbiota, probiotics and prebiotics. Cancer Lett. 2014;345:258–70. 23981580 10.1016/j.canlet.2013.08.013

[bib37] Shi C , Liang Y, Yang J et al. MicroRNA-21 knockout improve the survival rate in DSS induced fatal colitis through protecting against inflammation and tissue injury. PLoS One. 2013;8:e66814. 23826144 10.1371/journal.pone.0066814 PMC3691313

[bib38] Singh N , Shirdel EA, Waldron L et al. The murine caecal microRNA Signature depends on the presence of the endogenous microbiota. 2012;8:171–86. 10.7150/ijbs.8.171 PMC3248702 22211115

[bib63_1629379683762] Singla-Rastogi M . Charvin M Thiébeauld O et al. Plant Small RNA Species Direct Gene Silencing in Pathogenic Bacteria as well as Disease Protection. BioRxiv. 2019. DOI: 10.1101/863902.

[bib39] Soper T , Mandin P, Majdalani N et al. Positive regulation by small RNAs and the role of Hfq. Proc Natl Acad Sci USA. 2010;107:9602–7. 20457943 10.1073/pnas.1004435107 PMC2906882

[bib40] Suzuki K , Meek B, Doi Y et al. Aberrant expansion of segmented filamentous bacteria in IgA-deficient gut. Proc Natl Acad Sci USA. 2004;101:1981–6. 14766966 10.1073/pnas.0307317101 PMC357038

[bib41] Teng Y , Ren Y, Sayed M et al. Plant-derived exosomal microRNAs shape the gut microbiota. Cell Host Microbe. 2018;24:637–52. 30449315 10.1016/j.chom.2018.10.001 PMC6746408

[bib42] Thursby E , Juge N. Introduction to the human gut microbiota. Biochem J. 2017;474:1823–36. 28512250 10.1042/BCJ20160510 PMC5433529

[bib43] Timár CI , Lőrincz ÁM, Csépányi-Kömi R et al. Antibacterial effect of microvesicles released from human neutrophilic granulocytes. Blood. 2013;121:510–8. 23144171 10.1182/blood-2012-05-431114 PMC3548170

[bib44] Turchinovich A , Weiz L, Langheinz A et al. Characterization of extracellular circulating microRNA. Nucleic Acids Res. 2011;39:7223–33. 21609964 10.1093/nar/gkr254 PMC3167594

[bib45] Valadi H , Ekström K, Bossios A et al. Exosome-mediated transfer of mRNAs and microRNAs is a novel mechanism of genetic exchange between cells. Nat Cell Biol. 2007;9:654–9. 17486113 10.1038/ncb1596

[bib47] Van Niel G , Mallegol J, Bevilacqua C et al. Intestinal epithelial exosomes carry MHC class II peptides able to inform the immune system in mice. Gut. 2003;52:1690–7. 14633944 10.1136/gut.52.12.1690 PMC1773888

[bib46] Van Niel G , Raposo G, Candalh C et al. Intestinal epithelial cells secrete exosome-like vesicles. Gastroenterology. 2001;121:337–49. 11487543 10.1053/gast.2001.26263

[bib48] Veziroglu EM , Mias GI. Characterizing extracellular vesicles and their diverse RNA contents. Front Genet. 2020;11:700. 32765582 10.3389/fgene.2020.00700 PMC7379748

[bib61_1629378944715] Vickers KC , Palmisano BT, Shoucri BM et al. MicroRNAs are transported in plasma and delivered to recipient cells by high-density lipoproteins. Nat Cell Biol. 2011;13:423–33. 21423178 10.1038/ncb2210 PMC3074610

[bib49] Walters WA , Xu Z, Knight R. Meta-analyses of human gut microbes associated with obesity and IBD. FEBS Lett. 2014;588:4223–33. 25307765 10.1016/j.febslet.2014.09.039 PMC5050012

[bib50] Weber JA , Baxter DH, Zhang S et al. The microRNA spectrum in 12 body fluids. Clin Chem. 2010;56:1733–41. 20847327 10.1373/clinchem.2010.147405 PMC4846276

[bib51] Weiberg A , Wang M, Lin F-M et al. Fungal small RNAs suppress plant immunity by hijacking host RNA interference pathways. Science. 2013;342:118–23. 24092744 10.1126/science.1239705 PMC4096153

[bib52] Wen Li , Ley RE, Volchkov PY et al. Innate immunity and intestinal microbiota in the development of Type 1 diabetes. Nature. 2008;455:1109–13. 18806780 10.1038/nature07336 PMC2574766

[bib53] Wu CW , Ng SSM, Dong YuJ et al. Detection of miR-92a and miR-21 in stool samples as potential screening biomarkers for colorectal cancer and polyps. Gut. 2012;61:739–45. 21930727 10.1136/gut.2011.239236

[bib54] Wu F , Zikusoka M, Trindade A et al. MicroRNAs are differentially expressed in ulcerative colitis and alter expression of macrophage inflammatory peptide-2 alpha. Gastroenterology. 2008;135:1624–35. 18835392 10.1053/j.gastro.2008.07.068

[bib55] Wu H-J , Ivanov II, Darce J et al. Gut-residing segmented filamentous bacteria drive autoimmune arthritis via T helper 17 cells. Immunity. 2010;32:815–27. 20620945 10.1016/j.immuni.2010.06.001 PMC2904693

[bib56] Yang Y , Ma Y, Shi C et al. Overexpression of miR-21 in patients with ulcerative colitis impairs intestinal epithelial barrier function through targeting the Rho GTPase RhoB. Biochem Biophys Res Commun. 2013;434:746–52. 23583411 10.1016/j.bbrc.2013.03.122

[bib57] Yau TOn , Tang C-M, Harriss EK et al. Faecal microRNAs as a non-invasive tool in the diagnosis of colonic adenomas and colorectal cancer: a meta-analysis. Sci Rep. 2019;9:9491. 31263200 10.1038/s41598-019-45570-9 PMC6603164

[bib58] Yin Y , Cai X, Chen Xi et al. Tumor-secreted miR-214 induces regulatory T cells: a major link between immune evasion and tumor growth. Cell Res. 2014;24:1164–80. 25223704 10.1038/cr.2014.121 PMC4185347

[bib59] Zhang X , Zuo X, Yang Bo et al. MicroRNA directly enhances mitochondrial translation during muscle differentiation. Cell. 2014;158:607–19. 25083871 10.1016/j.cell.2014.05.047 PMC4119298

[bib60] Zhu K , Liu M, Fu Z et al. Plant microRNAs in larval food regulate honeybee caste development. PLos Genet. 2017;13:e1006946. 28859085 10.1371/journal.pgen.1006946 PMC5578494

